# Influence of point mutations on the stability, dimerization, and oligomerization of human cystatin C and its L68Q variant

**DOI:** 10.3389/fnmol.2012.00082

**Published:** 2012-07-27

**Authors:** Aneta Szymańska, Elżbieta Jankowska, Marta Orlikowska, Izabela Behrendt, Paulina Czaplewska, Sylwia Rodziewicz-Motowidło

**Affiliations:** Faculty of Chemistry, Department of Medicinal Chemistry, University of GdańskGdańsk, Poland

**Keywords:** cystatin C, mutants, stability, dimerization, oligomerization, crystal structure

## Abstract

Human cystatin C (hCC) is a small but very intriguing protein. Produced by all nucleated cells is found in almost all tissues and body fluids where, at physiological conditions, plays a role of a very potent inhibitor of cysteine proteases. Biologically active hCC is a monomeric protein but during cellular trafficking it forms dimers, transiently losing its inhibitory activity. *In vitro*, dimerization of cystatin C was observed for the mature protein during crystallization trials, revealing that the mechanism of this process is based on the three dimensional swapping of the protein domains. In our work we have focused on the impact of two proposed “hot spots” in cystatin C structure on its conformational stability. Encouraged by promising results of the theoretical calculations, we designed and produced several hCC hinge region point mutation variants that display a variety of conformational stability and propensity for dimerization and aggregation. A similar approach, i.e., rational mutagenesis, has been also applied to study the amyloidogenic L68Q variant to determine the contribution of hydrophobic interactions and steric effect on the stability of monomeric cystatin C. In this overview we would like to summarize the results of our studies. The impact of a particular mutation on the properties of the studied proteins will be presented in the context of their thermal and mechanical stability, *in vitro* dimerization tendency as well as the outcome of crystallization. Better understanding of the mechanism and, especially, factors affecting conformational stability of cystatin C and access to stable monomeric and dimeric versions of the protein opens new perspectives in explaining the role of dimers and the domain swapping process in hCC oligomerization, as well as designing potential inhibitors of this process.

## Introduction

More than 50 years after its discovery in human body fluids (Clausen, 1961) and 30 years after determination of its amino acid sequence (Grubb and Löfberg, 1982), cystatin C (hCC) still attracts the attention of many groups of scientists. Basic, unbiased search of the PubMed database yields over 2800 hits for “cystatin C” as a subject, most of them focused on clinical application of this protein as a valuable biomarker of various disease states. The original application of cystatin C as a measure of the glomerular filtration rate (Grubb et al., 1985) and a marker of kidneys function (Grubb, 1992; Page et al., 2000; Curhan, 2005) has expanded and now hCC is investigated also as a prognostic marker of cardiovascular events (Zethelius et al., 2008; Taglieri et al., 2009; Shin et al., 2012), as well as several forms of cancer (Turk et al., 2008). Another branch of the research is focused on the role of cystatin C in Alzheimer's disease, as both a biomarker and potential target for therapy (Levy, 2008; Zerovnik, 2009; Craig-Schapiro et al., 2011).

Less attention seems to be paid to cystatin C on a molecular level. Comparing to its homologues from the stefin family, stefins A and B, which are extensively studied by Turk, Zerovnik and co-workers (Zerovnik et al., 1998; Zerovnik and Kopitar-Jerala, 2006; Ceru et al., 2008; Zerovnik et al., 2010), the literature concerning cystatin C is much more scarce. Until last year the structure of the monomeric hCC was not determined (Kolodziejczyk et al., 2010; Orlikowska et al., 2011b), even though the biological activity of this protein is strictly dependent on its oligomeric state—only monomeric cystatin C is an active inhibitor of the papain-like cysteine proteases (Ekiel and Abrahamson, 1996). The best studied feature of cystatin C is its dimerization. Systematic studies of this process were initiated by Ekiel and Abrahamson (1996) and its mechanism was solved 5 years later, when it was shown to be based on the three dimensional domain swapping phenomenon (Janowski et al., 2001). Further studies, executed by Grubb and co-workers connected hCC ability to swap domains with its fibrillization propensities (Nilsson et al., 2004; Wahlbom et al., 2007). Interestingly, in the family of cysteine proteases inhibitors [I25, MEROPOS classification (Rawlings et al., 2012)], the propensity to exchange folding subunits is not limited only to cystatin C. Also members of the cystatin subfamily I25A—stefin A and B, as well as an avian cystatin C analogue (chicken cystatin, cC) also are able to undergo the same process. Intertwined dimers of stefin A and cC were shown to form upon nearly complete unfolding, resulting either from elevated temperature, or presence of relatively high concentration of denaturing agent (guanidinium chloride) and the structure of dimeric stefin A was confirmed by NMR studies (Jerala and Zerovnik, 1999; Staniforth et al., 2001). Domain-swapped dimer of Y31-stefin B P79S mutant was observed both in solution and in the crystal form as a part of a tetramer (Jenko Kokalj et al., 2007), in which two dimers interact with each other *via* the “hand shaking” mechanism, involving the exchange of two hairpin loop structures.

Special case of cystatin C dimerization refers to its naturally occurring point mutation variant, in which native leucine in position 68 is substituted by glutamine (Palsdottir et al., 1989). Caused by this mutation disruption of a network of the hydrophobic interactions, holding together the core of the protein, results in a strong destabilization of the molecule (Janowski et al., 2001). *In vitro* this feature of cystatin C variant is reflected by significantly increased tendency of L68Q hCC for dimerization and aggregation (Abrahamson and Grubb, 1994); *in vivo*—leads to massive formation of amyloid-like deposits of the protein in brain arteries and development of a lethal disease: hereditary cystatin C amyloid angiopathy (HCCAA; Palsdottir et al., 2006). The wild-type cystatin C is relatively stable in a monomeric form, however, it was shown that *in vivo* it undergoes transient dimerization during its post-translational trafficking and secretion (Merz et al., 1997). Whether the mechanism of this process is based on domain swapping was not investigated.

The relationships between cystatin C dimerization and oligomerization, localization, and activity are even more intriguing if one considers that most of the cysteine proteases are localized inside the cell, whereas hCC is an extracellular protein. Therefore, there must exist the mechanism that brings these molecules together, the more, that substantial and rapid hCC uptake was observed for some cancer cell lines (Ekström et al., 2008) and in neuroblastoma cells (Wallin et al., 2010). It is tempting to hypothesize that at some stages of cystatin C trafficking to and from the cell it adopts the inactive, dimeric, domain swapped form which, later on, undergoes monomerization and activation in the response to increased levels of its target proteases.

Regardless the substantial efforts and many experimental and theoretical studies aimed at developing a comprehensive model for the domain swapping, this process still remains quite elusive and very case-sensitive. Large structural and sequence diversity of proteins undergoing the domain swapping process does not simplify the efforts (Shameer et al., 2011). However, there are some conclusions that were reached, focusing the attention of the researchers on two main molecular factors that may be involved in increased protein's propensity to swap domains. First of them are local strains arising from the presence of unfavorable amino acid sequence (Sirota et al., 2008), whereas the second one points to the crucial role of flexible hinge regions in the protein structure (Dehouck et al., 2003; Yang et al., 2004; Ding et al., 2006; Gronenborn, 2009). Human cystatin C (hCC) is a unique protein that allows studies of the impact of both these factors on the domain swapping, dimerization, and oligomerization.

## Intramolecular determinants of cystatin C domain swapping process

**Figure 1:**
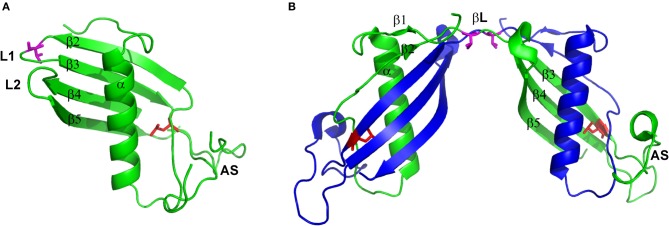
**Structure of (A) hCC monomer (stab1, PDB code: 3GAX) and (B) dimer (PDB code: 1G96).** Valine residue in position 57 presented as magenta sticks and leucine in position 68 presented as red sticks. Figure prepared using PyMol software.

Cystatin C is a small, basic protein, which 120 amino acids long polypeptide chain is folded to create a compact, fist-like molecule, with a helical “thumb” wrapped by four “fingers” created by anti-parallel β-strands (Kolodziejczyk et al., 2010; Figure 1A). Two β-hairpin loops, L1 and L2, localized on the one pole of the molecule form, together with the N-terminal part, the inhibitory active site recognized by the main target of all cystatins—cysteine proteases. The opposite, mainly unstructured, appending structure (AS) contains the region recognized by the other group of enzymes—legumains (Alvarez-Fernandez et al., 1999). The loop L1 is also vitally engaged in cystatin C dimerization. It joins two hCC fragments being exchanged in the domain swapping: the N-terminal β1-α−β2 fragment and C-terminal β2-AS-β4-L2-β5 part (Jaskolski, 2001; Figure 1B). It is also the only structural element that undergoes significant change during the domain swapping process, turning from bent to extended conformation. Such features make loop L1 a model example of the so-called “hinge region.” Programs devoted to prediction of flexible protein fragments, potentially involved/responsible for the domain swapping such as HingeProt, Fugue, or PoPMuSiC (Ding et al., 2006; Emekli et al., 2008) point to cystatin C fragments centered around residues 43–59 as flexible and having preferences for non-native conformation. Residues Q^55^IVAG^59^ forming loop L1 coincide with this prediction.

The second factor, significantly shifting the hCC monomer–dimer ratio toward the dimer is unique to the natively occurring cystatin C point mutant in which, due to single-nucleotide mutation, native leucine is substituted by glutamine. Introduction of a polar and sterically bigger side chain into the hydrophobic core of the protein, at the interface between two labile domains (Figures 1A,B), significantly destabilizes the molecule and, with the aid of the molecular tensions within the hinge loop L1, leads to pronounced dimerization and aggregation at physiological conditions. Soluble hCC L68Q oligomers can be detected in body fluids, but aggregation of this protein primarily affects the brain (Olafsson et al., 1996). Accumulation of hCC L68Q deposits in brain arteries leads to development of an amyloidogenic disease—HCCAA, which is manifested by numerous hemorrhages, strokes, and finally death, often before 40 years of age (Palsdottir et al., 2006).

## The sequence of the hinge loop as a gatekeeper of the wild-type cystatin C conformational stability

As it was mentioned above, the hCC loop L1 fulfills the requirements expected for the effective hinge region in the domain swapping process. Comparison of its sequence with the data for other cystatins available currently in the PDB database, and further analysis in the context of the torsional φ and ψ angle values revealed, that there is the amino acid residue displaying strained conformation, namely valine in position 57, located near the apex of the loop (Rodziewicz-Motowidło et al., 2009; Figure 1A). These tensions may be important for the biological activity of cystatin C, but they may also account for its increased dynamics, especially in the absence of the target protease. Therefore, we decided to perform theoretical studies in order to assess the possibility of stabilization of the cystatin C hinge loop by amino acid substitution. Molecular dynamics calculations were performed for two mutations expected to stabilize (V57D and V57N) and one that should additionally broaden the loop L1 (V57P) and increase the tendency of hCC for opening (Rodziewicz-Motowidło et al., 2009). Even though the mechanism of the domain swapping, not only for cystatin C, but also for other proteins, is still being discussed, there is an agreement that the entire process must be preceded by at least partial unfolding of the molecule (Liu et al., 2007). The most probable places of such unfolding are flexible hinge regions, which, due to their unique, molecular spring-like properties, may facilitate the unfolding process. Therefore, all changes designed to stabilize or at least limit the dynamics of the crucial fragment should lead to stable monomeric form of the protein. The calculations performed for hCC fragments encompassing β-hairpin formed by β2-L1-β3 fragment (His43-Thr72) revealed, that even though for all studied peptides the broadening of the structure was observed, introduction of favored in turns Asp and Asn residues increased conformational stability of the analyzed hCC fragment. On the other hand, the V57P loop L1 variant was shown to exist in two conformational states, separated by small, about 2 kcal/mol, free energy barrier (Rodziewicz-Motowidło et al., 2009).

To verify the results of our calculations we have produced the full length cystatin C variants with the appropriate mutations introduced by means of site directed mutagenesis (Szymańska et al., 2009). To our satisfaction, all mutants turned out to be stable and soluble proteins, properly trafficked to the periplasmic space during bacterial over-expression and easy to isolate and purify in high yields. Moreover, the cold osmotic shock isolation and subsequent ion-exchange purification of the V57P mutant yielded the protein as a dimer/monomer mixture, exactly as we expected based on the molecular dynamics calculations results. The asparagine and aspartic acid hCC counterparts at the same conditions rendered monomeric.

Structural studies revealed that the introduced mutations do not change significantly the secondary and tertiary structure of the studied hCC variants. Circular dichroism measurements performed in the far-UV range confirmed that all proteins display predominantly α/β fold. However, lower values of the molar residual ellipticity observed for V57D and V57N variants, especially at 208 nm, suggest higher content or stability of the α-helical part for these proteins. The content of the secondary structure elements in proline mutant seems to be lower, comparing to other studied proteins (including the wild-type hCC), but this difference may result from different oligomeric (dimeric) state of this variant. Similar fold of hCC hinge loop variants, including dimeric V57P, was also confirmed by the near-UV CD (Szymańska et al., 2009). Minor differences, concerning the position of a minimum of the spectra, may be attributed to the form (monomeric vs. dimeric) of the investigated molecules in solution, rather than significant differences in their structure. Structural similarity of the proline variant, however, did not correlate with its thermal stability (Jankowska et al., 2008). Thermal unfolding of hCC V57P, monitored by CD measurements at two wavelengths (218 and 222 nm) revealed that, regardless the wavelength of observation, the measured Tm = 71°C for this protein is lower by 10°C than for other hinge loop mutants (Jankowska et al., 2004, 2008). In contrast to the wild-type hCC, proline, and two other variants unfold in more cooperative manner and only one transition is observed at both wavelengths. For native cystatin C two transitions can be observed at 222 nm, one at 75°C and one at 84°C. Taking into account that dimeric hCC predominates in solution at temperatures in the range 65–80°C (Ekiel and Abrahamson, 1996), the first transition may depicts unfolding of already dimeric, but not necessarily the domain-swapped protein. Formation of such dimer must be preceded by more pronounced changes in the hCC structure, involving separation of the entire N-terminal domain, containing also the helical part from the core of the molecule. It is not very likely that such change would not be visible in the CD spectrum. Therefore, it may be hypothesized that the first unfolding reflects changes leading to the formation of the domain-swapped molecule, which next unfolds at higher temperature. Lowered thermal stability of the hCC α-helical part suggests that the conformational changes leading to cystatin C unfolding the most likely start at this region of the protein, and that this structural element is important for the overall conformational stability of the cystatin C.

The predicted increased or decreased stability of hCC variants in the monomeric form was further studied by monitoring their susceptibility to undergo dimerization induced by denaturating agents. According to Ekiel, moderate concentration of guanidine hydrochloride in the assay buffer accelerates significantly the rate of the wild-type cystatin C dimerization *in vitro*. Therefore, we have tested the behavior of all proteins during prolonged incubation in the presence of 0.5 and 1.0 M GdnHCl in PBS buffer (pH 7.4; Szymańska et al., 2009). Comparing to the reference, the native cystatin C, very low degree of dimerization was observed for the V57D and V57N variants at both studied conditions (Table 1). The V57P mutant retained its dimeric state during the incubation in the presence of lower concentration of the destabilizing agent, whereas at 1.0 M GdnHCl concentration some increase in the monomer fraction was observed, suggesting comparable stability of both forms at these conditions.

**Table 1:** **Dimer content (%) formed by cystatin C and its hinge loop mutants incubated in the presence of different concentration of guanidinium chloride (GdnHCl; Szymańska et al., 2009)**.

	**Conditions**					
	**PBS, pH 7.4**		**PBS, pH 7.4**		**PBS, pH 7.4**	
			**0.5 M Gdn HCl**		**1.0 M Gdn HCl**	
	**Start**	**9 days**	**Start**	**9 days**	**Start**	**9 days**
hCC wt	3.30	0.20	6.42	31.43	77.22	77.46*
hCC V57D	2.14	2.78	1.02	8.13	9.34	15.72
hCC V57N	4.56	2.70	4.49	7.68	1.94	6.93
hCC V57P	80.40	64.83*	88.21	88.71	50.27	63.02

* Precipitation of the protein observed.

To further confront the results of our biochemical studies concerning in-solution stability of hCC V57 mutants with the proteins' structure, we performed crystallization trials. The V57N mutant occurred to be stable as a monomer and crystallized in a monomeric form (Orlikowska et al., 2011b). The structure of the mutant follows the cystatins' general fold and consists of a five-stranded antiparallel β-sheet wrapped around a long central α-helix (PDB code: 3NX0). In the C-terminal part of the molecule, two characteristic disulfide bridges are observed. Comparison of hCC V57N and the dimeric, domain-swapped structure of the wild-type cystatin C, revealed by Janowski et al. (2001), confirmed that the general fold of the monomeric cystatin molecule is duplicated in the structure of the dimeric hCC. The only exception constitutes the L1 loop which undergoes straightening during the domain swapping event and becomes a part of an unusually long β-sheet, formed by two copies of strands Tyr42-Thr74 (βL on Figure 1B). Interestingly, substitution of the strained valine residue with asparagine, regardless the stabilization of the protein in the monomeric form, does not seem to ease significantly the tensions within the loop L1. Comparison of hCC V57N crystal structure with the published in 2010 first crystal structure of the monomeric variant of hCC (Kolodziejczyk et al., 2010), stabilized against domain swapping by “stitching” the exchanging domains using the engineered disulfide bridge (Cys47-Cys69), reveals high similarity of both loops (and the whole molecules as well). The most significant distortions occur at the dihedral angles of Val57 and Asn57 residues, placing them, respectively, in additionally or generously allowed regions of the Ramachandran plot and suggesting the strained conformation as an inherent feature of the amino acid occupying this position in hCC molecule. Similarity of all the remaining protein segments is an experimental proof that the structural perturbations accompanying the dimerization process involve mainly or even exclusively the L1 region. It provides also a strong support for the hypothesis that the dimerization proceeds through relaxation and opening of hCC structure, in which process the L1 loop plays an important role.

Replacement of Val57 with aspartic acid was also intended to preserve the L1 loop structure and prevent hCC dimerization. We anticipated a reduced tendency for the dimer formation basing on the fact that, similarly to asparagine, aspartic acid residue is frequently spotted in turn regions and it is known to stabilize such conformations. Our expectations were also supported by the results of the experiments performed on cC bearing V55D mutation (equivalent to V57D in the human counterpart), which showed the mutated protein as a monomer under all conditions used to dimerize the wild-type cC (Staniforth et al., 2001). Aspartic acid in the hinge loop of hCC, however, did not satisfy the expectations in all respects. Although in chemical and thermal denaturation processes V57N and V57D mutants demonstrated significant and comparable stability of their monomeric forms (Szymańska et al., 2009), in the crystal the V57D variant appeared as a domain-swapped dimer (Orlikowska et al., 2010a, 2011a), closely resembling the crystal form of the wild-type cystatin C. The only difference between V57N and V57D mutants is the character of the side chain of the amino acid replacing the residue 57. While this chain is merely polar in asparagine residue, in aspartic acid at pH above 3.9 it takes a form of a carboxylate anion. Insertion of the charged residue in the middle of the extended β-sheet created by β2 and β3 strands in the dimeric molecule may be expected to diminish the structure stability due to repulsive interactions, and hence, should shift the equilibrium toward the monomer. Preference of hCC V57D to exist as the domain-swapped dimer suggests, that the spatial arrangement of the side chain of D57 residue does not introduce repulsive interactions. On the contrary, conformational change resulting from the dimerization might enable more favorable contacts of aspartic acid side chain with the solvent molecules. These contacts might be a source of the enthalpic gain stabilizing the dimer.

Proline is regarded as a residue which may play a pivotal role in modulating domain swapping propensity in some proteins (Bergdoll et al., 1997; Rousseau et al., 2001). We have decided to introduce proline into the critical 57 position of hCC molecule to test its power to induce the turn broadening and influence hCC tendency to swap domains. In the case of this mutant results of the denaturation studies went in line with the crystallization experiments. In each tested condition V57P displayed enhanced propensity to dimerization (Szymańska et al., 2009) and in the crystal it was also a dimer (PDB code: 3S67; Orlikowska et al., 2012). Rigidity of the proline cyclic side chain probably plays a crucial role in the monomer destabilization causing escalation of the backbone strain in the L1 loop region. The increased strain makes this loop act as a loaded molecular spring that relieves tension by adopting an alternative—extended conformation, triggering partial unfolding of the protein molecule and subsequent dimerization with domain swapping. Additionally, among the studied cystatin C hinge loop variants, V57P exhibits highest propensity for self-association, both in crystal (Orlikowska et al., submitted) and in solution.

Our approach toward stabilization of hCC in the monomeric form, based on minimized structural intervention, limited to a single-point mutation within the hinge region, turned out to be partially successful. The obtained monomeric structure for V57N and dimeric for V57P mutant confirmed validity of our strategy. On the other hand, the dimeric structure of V57D variant taught us a humility lesson. To our consolation, we have designed and very recently resolved the crystal structure of yet another monomeric cystatin C hinge loop variant (data not published). Replacement of the critical valine residue with the simplest amino acid—glycine, yielded exceptionally stable monomeric protein, showing very low dimerization propensity in the *in vitro* tests (Behrendt, 2010). The V57N and the V57G monomeric structures are together an indication that it is possible to influence the protein dimerization propensity by manipulation in the sequence of the hinge region, even if the scope of this manipulation is limited only to a single-point mutation.

## Immutability of the hydrophobic interior essential to cystatin C stability

We have also studied the impact of the second “hot spot” in the hCC sequence on its conformational stability. As it was mentioned above, the naturally occurring L68Q cystatin C variant shows not only increased dimerization propensity (Nilsson et al., 2004) but is also highly amyloidogenic (Abrahamson and Grubb, 1994). As it was proposed by Janowski, polar and sterically bigger glutamine residue, introduced into the hydrophobic core of the molecule, can easily disrupt the network of hydrophobic interactions stabilizing the α/β interface, triggering unfolding and subsequent domain swapping (Janowski et al., 2001). Another factor, accounting for increased dimerization propensity of the hCC L68Q variant may be lowering of the unfolding energy barrier due to reduction of the unfavorable solvent contacts in the unfolded (or partially unfolded), more polar cystatin C variant comparing to the wild-type protein. Results of our theoretical calculations, performed on model, monomeric forms of both proteins confirmed fully above hypotheses and brought some additional information about other consequences of the introduced mutation (Rodziewicz-Motowidło et al., 2006). As expected, in the amyloidogenic hCC variant the distance between the α-helix and the β-sheet protein core is bigger by c.a. 0.4 Å (9.13 Å for wt vs. 9.55 Å for L68Q variant). As a result, the variant is less compactly folded and displays increased flexibility of the exchangeable protein fragments. Expanding of the mutated protein molecule leads to increased solvent accessibility (by 6%) and to weakening of the hydrogen bonds and salt bridges network. Additionally, 23 kcal/mol difference of the non-bonded energy value, calculated for the wild-type and L68Q cystatin C was proposed by us as an additional driving force of the dimerization process for the later protein (Rodziewicz-Motowidło et al., 2006).

In order to assess the impact of both, electrostatic and steric, forces on cystatin C conformational stability in solution (similarly to the loop L1) we designed additional hCC variants, in which position 68 was occupied with hydrophobic but bigger than leucine phenylalanine, hydrophobic and smaller valine and isosteric to the native leucine but polar asparagine residue, respectively. Among these proteins, only L68V mutant turned out to be soluble and could be obtained in satisfactory yields from the *Escherichia coli* periplasmic space, similarly to the hinge loop variants. The other mutants, together with L68Q as a reference, even though expressed in high yields, turned out to be contained into inclusion bodies. All purification efforts yielded only low milligram amounts (1–3 mg) of proteins, with high propensity for self-association and aggregation, which made more detailed analyses impossible.

The only soluble and stable hydrophobic core variant of hCC, L68V, could be purified in the monomeric form, but, comparing to the wild-type cystatin C, it shows increased tendency for dimerization (Orlikowska et al., 2010b), even under milder destabilization conditions. This feature places hCC L68V variant between the wild-type protein and the amyloidogenic L68Q mutant, suggesting at the same time that the hydrophobic interactions within the core of the hCC molecule are crucial for its stability. High instability of the L68N hCC, comparable with destabilization observed for the hCC L68Q and caused by the isosteric but polar asparagine residue taking the place of the native leucine, supports this hypothesis. In this case the steric effect can be considered as negligible and the observed intramolecular destabilization must arise from the disruption of hydrophobic interface between the α-helix and the β-sheet core of the molecule, triggering its opening and subsequent dimerization/oligomerization.

**Figure 2:**
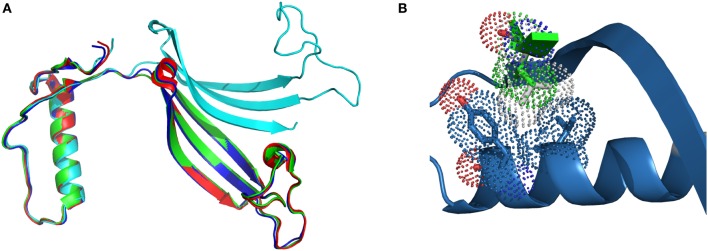
**Comparison of (A) the crystal structure of hCC wt (red, PDB code: 1G96) and its variants: L68V (green—pH 4.0, PDB code: 3PS8; cyan—pH 8.0, PDB code: 3QRD) and V57P (blue, PDB: 3S67); (B) molecular contacts of Val68 (van der Waals radius as green dots) and Leu68 (gray dots) residues.** Figure prepared using PyMol software.

In the case of L68V, the interactions between the exchangeable hCC domains are weakened due to the smaller van der Waals radius of Val residue, but not to the point of changing the overall fold of the protein. Successful crystallization trials, performed at two different conditions: pH 4.6; PDB code 3PS8, and pH 8.0; PDB code 3QRD (Orlikowska et al., 2010b) yielded the domain-swapped dimer, similar to the ones observed for the wild-type protein and the two hinge loop hCC variants (Figure 2A). Closer examinations of the surroundings of the substituted position 68 (Figure 2B) shows that the distance between the α-helix and the β-sheet is the same in both dimers. Therefore, lowered stability of the hCC L68V variant in the monomeric form cannot be attributed to changes in the secondary or tertiary structure of the studied protein, but, as proposed above, impaired interactions between the α-helix and the C-terminal β-sheet.

The results obtained for the hydrophobic core hCC mutants clearly indicate, that the integrity of the hydrophobic core of hCC is crucial for its conformational stability. Introduction of polar, not to mention charged, residue destabilizes the protein molecule to the point of rendering it amyloidogenic. Higher, in relation to L68Q and L68N, but lower, when compared with the hinge loop mutants, stability of hCC L68V variant strengthens this hypothesis, showing at the same time, that changes in this hCC region are not well tolerated. The importance of the hydrophobic interactions can be further supported by the observation, that the deleterious effect of introduction of a polar glutamine residue cannot be alleviated by stabilization of the hinge loop (Biernacka, 2010). Double hCC variants, containing both the L68Q and V57D or V57N mutations, turned out to be either expressed in very low yield or, due to either impaired cellular trafficking and/or folding, easily degraded *in vivo*. Minute amounts obtained from the inclusions bodies mimicked rather the properties of the hydrophobic core, than the hinge loop variants displaying low thermal stability and high propensity to first dimerization and next precipitation.

## Discussion

### The impact of the mutations in the hinge loop on oligomerization of cystatin C

**Figure 3:**
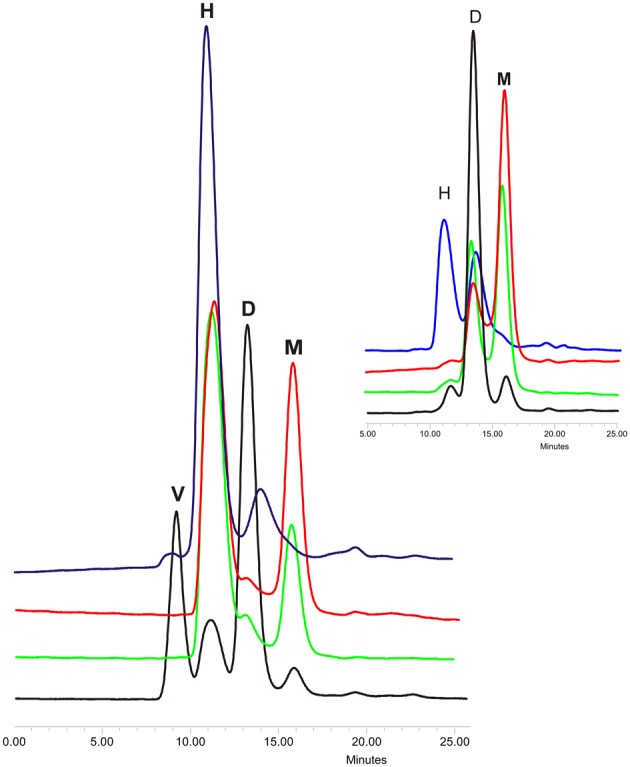
**Gel filtration of oligomerized hCC wt (black) and its hinge loop mutants: V57D (green), V57N (red), and V57P (blue).** Protein samples were incubated in 50 mM AcONa (pH 4.0), 100 mM NaCl at 48°C for 2 h with constant mixing and subjected onto Superdex 75 column equilibrated in the same buffer. In the inset chromatograms of the protein samples before incubation are presented. Peak descriptions: **M**, monomeric hCC; **D**, dimeric (domain swapped) hCC; **H**, putative hexameric hCC assembly; **V**, higher hCC oligomers eluted in the void volume of the column. The SEC elution profiles are given for a better illustration of the data described in Szymańska et al. (2008).

Proteins from cystatin family are counted among the amyloidogenic ones (Staniforth et al., 2001). This description stands for both stefins (Zerovnik et al., 2010) and for cystatin C (especially its L68Q variant) (Janowski et al., 2001). The mechanism of proteins oligomerization and fibrillization is still being discussed, especially the transformation of the soluble oligomeric species into mature fibrils (Uversky, 2010) and the role and structure of the oligomers and folding intermediates in the aggregation process (Jahn and Radford, 2008; Skerget et al., 2009; Stefani, 2012). To make the picture more complex, there are also proposed different models of proteins' ordered aggregation into fibrillar structures (Zerovnik et al., 2011) among which the domain swapping mechanism is being considered as the one involved in fibrillization of, e.g., prion proteins (Hafner-Bratkovič and Jerala, 2011) and cystatins (Wahlbom et al., 2007; Zerovnik et al., 2011). The direct proof for the connection between these two processes in the case of cystatin C was provided by Wahlbom and coworkers, who has shown, that inhibition of the domain swapping by covalent linking of the exchanging protein's parts abolishes also its fibrillization. Having in hands other cystatin C mutants with modulated dimerization tendency, we decided to check also their tendency for further oligomerization (Szymańska et al., 2008). According to Wahlbom, cystatin C incubated in an acidic buffer (pH 4.0) at elevated temperature and with continuous agitation forms doughnut-shaped oligomeric forms (Wahlbom et al., 2007) via propagated domain swapping (“run-away”) mechanism. The hCC hinge loop mutants were also subjected to similar studies. Protein samples were incubated at conditions promoting oligomerization for 2 h and the results were checked using gel filtration on Superdex 75 column equilibrated in the assay buffer. The analysis of the obtained data revealed that all studied proteins are capable of forming oligomers higher than dimers (Figure 3). For all three analyzed mutants the main oligomeric form had retention time about 11 min which, based on the column calibration, is expected for the hexameric assembly of the protein (Figure 3, peak H, MW ca. 80 kD). The highest content of monomers in the incubated samples was observed for the V57N protein, which was shown to be the least dimerization-prone. In the case of the initially dimeric proline mutant the majority of the protein was in the form of a putative hexamer. Interestingly, significant amount of the dimeric form was observed only for the native hCC, for which the H peak was much lower than for the other studied proteins. Additionally, for the wt protein, formation of higher oligomers was observed (Figure 3, peak V). The exact molecular weight of this specimen could not be determined due to its elution in the void volume of the SEC column used in this experiment. Application of the column with higher exclusion limit (Superdex 200) also did not bring reliable information.

**Figure 4:**
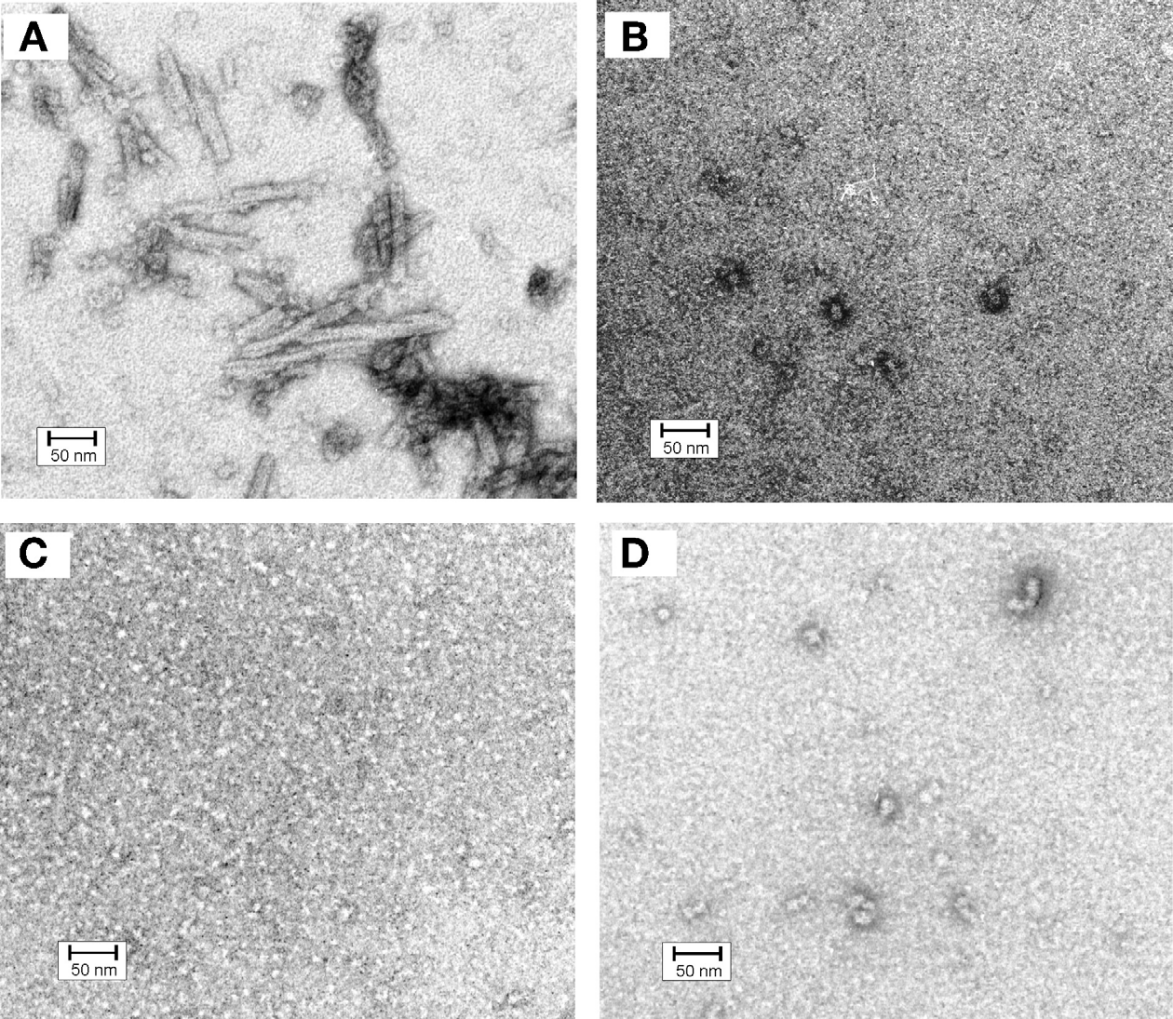
**Electron micrographs of cystatin C oligomers (A) HCC wt, (B) HCC V57D, (C) HCC V57N, and (D) HCC V57P.** Oligomers were produced by incubation of protein samples at 3 mg/ml concentration in 50 mM AcONa (pH 4.0), 100 mM NaCl, with continuous agitation at 48°C for 2 h. Bar representing 50 nm is shown as an indicator of the size. Protein samples were negatively stained with 2% (w/v) aqueous uranyl acetate for 30 s and examined with a Philips CM10 electron microscope (Philips, Eindhoven, The Netherlands), operating at an excitation voltage of 100 kV. The EM data were presented on a conference and were described but not reproduced in Szymańska et al. (2008). Here, the image (unpublished before) is given in order to enable better discussion.

In order to get a deeper insight into oligomerization of hCC variants all samples subjected to gel filtration were also inspected under the electron microscope (Szymańska et al., 2008). For the V57D and V57N variants the presence of any defined structural form was not observed (Figures 4B,C). The proline mutant formed at the same conditions few circular and irregular aggregates (Figure 4D), whereas for the native protein a new type of a structural object was observed (Figure 4A). In addition to doughnut-shaped oligomers reported previously by Wahlbom and co-workers, electron micrographs recorded after 2 h of incubation demonstrated rod-like structures with the outer diameter of 17 ± 2 nm and length of 100 ± 20 nm. The population of these oligomers seems to be quite homogeneous. The size of both rods and “doughnuts” is slightly bigger than the ones observed before. We hypothesize that the rod-like specimen may correspond to the pre-fibrillar intermediate of hCC formed by the protein undergoing the domain swapping in the propagated manner (“run-away” domain exchange model), “caught” at the point before it transforms to insoluble fibrils. The other possibility, which we cannot rule out, is that the observed rods are constituted of the “doughnuts” stacked on each other. Dimensions of the observed specimen, that is the outer diameter bigger than the diameter of the typical amyloid fibril and much shorter length, suggest that, in such a case, additional conformational change would be necessary for the mature fibril formation. Additionally, these structures did not display any increase in ThT fluorescence observed for amyloid fibrils (data not shown).

The lack of analogous structures for the rest of the studied hCC variants may suggest the important role of the domain swapping capability of a particular protein in their formation. Additionally, the lack of the rod-like oligomers for the hCC V57P, which is predominantly dimeric, may strengthen the hypothesis of the propagated manner of the domain exchange process. The dimer formed by this variant may be too stable to undergo opening and further oligomerization in the “run-away” mode.

## Conclusions and further directions

The results obtained for two groups of cystatin C variants: the hinge loop and the hydrophobic core mutants, show that the main stabilization force of the hCC molecule comes from the hydrophobic interactions responsible for holding together the α-helix and the C-terminal part of the molecule. Changes within these regions increase the propensity of the hCC toward dimerization and oligomerization, which cannot be easily compensated by stabilization of the hinge loop sequence. However, in the case of the wild-type protein, its stability in biologically active, monomeric form may be easily manipulated by changes in the hinge region sequence. The access to stable monomeric and dimeric forms of cystatin C, with marginally changed primary structure and available in high amounts is beneficial from the point of view of more detailed studies of the mechanism of the domain swapping in cystatin C as well as the implication between domain swapping, dimerization and oligomerization. Our approach based on the rational mutagenesis yielded a pool of data that encourages us to perform further studies of the hCC dimerization mechanism, especially of the early stages of this process and the role of the helical part. The next steps are therefore the folding studies of all obtained cystatin C variants and dissecting the mechanism of their functional or aberrant outcome leading to protein fibrillization. The knowledge concerning thermodynamics and kinetics of the hCC folding, especially the number and nature of intermediates, should provide the information crucial for better understanding of hCC aggregation process. Additionally, in the future work we intend to compare the folding mechanism and fibrillization propensities of the cystatin C mutants with the data obtained for other amyloidogenic proteins, not only stefins (Jenko et al., 2004; Kenig et al., 2006; Jelinska et al., 2011). The outcome of such studies could be used to verify the hypothesis about the connection between protein stability, the folding mechanism and fibrilization propensities.

### Conflict of interest statement

The authors declare that the research was conducted in the absence of any commercial or financial relationships that could be construed as a potential conflict of interest.
